# A Fibrin Coating Method of Polypropylene Meshes Enables the Adhesion of Menstrual Blood-Derived Mesenchymal Stromal Cells: A New Delivery Strategy for Stem Cell-Based Therapies

**DOI:** 10.3390/ijms222413385

**Published:** 2021-12-13

**Authors:** Federica Marinaro, Joana M. Silva, Alexandre A. Barros, Ivo M. Aroso, Juan C. Gómez-Blanco, Isaac Jardin, Jose J. Lopez, María Pulido, María Ángeles de Pedro, Rui L. Reis, Francisco Miguel Sánchez-Margallo, Javier G. Casado, Esther López

**Affiliations:** 1 Stem Cell Therapy Unit, Jesús Usón Minimally Invasive Surgery Centre, 10071 Cáceres, Spain; cargoblan15@gmail.com (J.C.G.-B.); mpulido@ccmijesususon.com (M.P.); madepedro@ccmijesususon.com (M.Á.d.P.); elopez@ccmijesususon.com (E.L.); 2 3B’s Research Group, I3Bs-Research Institute on Biomaterials, Biodegradables and Biomimetics, University of Minho, Headquarters of the European Institute of Excellence on Tissue Engineering and Regenerative Medicine, Avepark, Zona Industrial da Gandra, 4805-017 Barco, Guimarães, Portugal; joana.marques@i3bs.uminho.pt (J.M.S.); ip@i3bs.uminho.pt (A.A.B.); ivo.aroso@i3bs.uminho.pt (I.M.A.); rgreis@i3bs.uminho.pt (R.L.R.); 3 ICVS/3B’s-PT Government Associate Laboratory, 4805-017 Guimarães, Portugal; 4 Cell Physiology Research Group, Department of Physiology, University of Extremadura, 10003 Cáceres, Spain; ijp@unex.es (I.J.); jjlopez@unex.es (J.J.L.); 5 Institute of Molecular Pathology Biomarkers, University of Extremadura, 10003 Cáceres, Spain; jgarcas@unex.es; 6 Centro de Investigación en Red en Enfermedades Cardiovasculares (CIBERCV), 28029 Madrid, Spain; 7 Immunology Unit, Department of Physiology, University of Extremadura, 10003 Cáceres, Spain

**Keywords:** surgical mesh, fibrin sealant, coating, fibrin, cell therapy, menstrual blood-derived stromal cells

## Abstract

Polypropylene (PP) mesh is well-known as a gold standard of all prosthetic materials of choice for the reinforcement of soft tissues in case of hernia, organ prolapse, and urinary incontinence. The adverse effects that follow surgical mesh implantation remain an unmet medical challenge. Herein, it is outlined a new approach to allow viability and adhesion of human menstrual blood-derived mesenchymal stromal cells (MenSCs) on PP surgical meshes. A multilayered fibrin coating, based on fibrinogen and thrombin from a commercial fibrin sealant, was optimized to guarantee a homogeneous and stratified film on PP mesh. MenSCs were seeded on the optimized fibrin-coated meshes and their adhesion, viability, phenotype, gene expression, and immunomodulatory capacity were fully evaluated. This coating guaranteed MenSC viability, adhesion and did not trigger any change in their stemness and inflammatory profile. Additionally, MenSCs seeded on fibrin-coated meshes significantly decreased CD4+ and CD8+ T cell proliferation, compared to in vitro stimulated lymphocytes (*p* < 0.0001). Hence, the proposed fibrin coating for PP surgical meshes may allow the local administration of stromal cells and the reduction of the exacerbated inflammatory response following mesh implantation surgery. Reproducible and easy to adapt to other cell types, this method undoubtedly requires a multidisciplinary and translational approach to be improved for future clinical uses.

## 1. Introduction

The synthetic thermoplastic polymer polypropylene (PP) was firstly produced by Hogan and Banks in 1951 through the “Phillips catalyst” [1]. Due to its inertness, hardness, rigidity, strength, and resistance to acids, bases, and solvents, PP has been used in many non-medical and medical applications [2]. A variety of medical devices, as membrane oxygenators [3], syringes [4], sutures [5], and surgical meshes [6], have been produced with PP over the years.

In the case of surgical meshes, many absorbable and biological materials have been optimized for soft tissue reinforcement. However, due to their physical properties and biocompatibility, non-absorbable plastics, as PP, are still the materials of choice for the treatment of hernia, urinary incontinence, and pelvic organ prolapse [7]. In the past years, the use of meshes for stress urinary incontinence and pelvic organ prolapse has been strongly discouraged due to the unbalanced risk-benefit ratio [8]. Conversely, herniorrhaphy and hernioplasty using non-absorbable surgical meshes are still the gold standard treatments for ventral and groin hernias, leading to a substantial reduction in their recurrence [9]. However, PP mesh implantation for hernia repair has also been related to many adverse effects, like pain, foreign body rejection, adhesion to visceral organs, and mesh incorporation or encapsulation, followed by a reduction in mesh mechanical properties and infection [10]. Over time, researchers have developed a variety of PP mesh-coating methods to counteract these adverse effects, such as titanium [11], polycaprolactone and gelatin [12], chitosan and PLGA [13], methacryloyl gelatin and methacryloyl mucin [14], dopamine-mediated zwitterionic polysulfobetaine methacrylate [15], silk fibroin [16], extracellular matrix [17], and fibrin gel [18], among others. Fibrin gels are usually prepared from fibrin sealants, commonly used for mesh fixation [19]. Fibrin sealants are commercially available as separately packaged human fibrinogen and thrombin. Once combined, the final stage of the blood coagulation cascade is initiated and a fibrin gel-like compound is formed. Fibrin gels have been proposed by different authors as a vehicle to combine cell-based therapies and surgical mesh repair. In a previous study from our group, bone marrow-derived mesenchymal stromal cells (BM-MSCs) in culture medium were mixed with thrombin (500 IU/mL) at 1:1 ratio. This suspension was spread with fibrinogen (80 mg/mL) at a ratio of 1:1 on a PP mesh by using a standard fibrin glue applicator. This technique allowed us to fix the meshes in a murine model of incisional hernia and demonstrated the immunomodulatory potential of these bioactive meshes [20]. In another work from our group, porcine BM-MSCs were resuspended in a 3:1 ratio of cell culture medium to thrombin solution (500 IU/mL) and applied on the top of PP meshes with fibrinogen (500 mg/mL) with a fibrin glue applicator. These meshes were implanted in pigs with congenital abdominal hernia but did not lead to an improvement of hernia-related complications [21]. More recently, Guillaume et al. resuspended stromal vascular fraction (SVF) cells in a 4:1 ratio of PBS to fibrinogen. This suspension was quickly mixed with thrombin at ratio 5:2 and pipetted onto PP titanized meshes. The so-prepared meshes were implanted in immunodeficient rats with an experimentally created hernia defect and had an impact on angiogenesis of the abdominal wall [18]. 

Blázquez et al. [20] and Guillaume et al. [18] repaired small defects in murine models and they did not evidence any limitation in their studies. However, the use of large animal models is necessary to translate the results of research to the human clinical setting, so in our last study in this field [21], we investigated the effect of fibrin-coated meshes in a relevant porcine model of congenital hernia. Even though the coating method was similar to the other two studies [18,20], we noticed that spreading the suspension of cells and fibrin gel on the mesh was challenging, in terms of coating adhesion and uniformity.

Hence, with this work, we aimed to improve the fibrin coating method to ensure a better covering of PP mesh surface and a satisfactory adhesion of stromal cells. First, we functionalized the PP surgical meshes with different plasma treatments to improve PP hydrophilicity and adhesion abilities towards the fibrin coating. Second, various fibrin coating procedures for the PP meshes were tested. Third, the biological performance of the optimized fibrin-coated meshes was evaluated in terms of cell adhesion, viability, and immunomodulatory capacity. In conclusion, our study demonstrated that multilayered fibrin coatings of PP mesh, performed with dilutions of 5 mg/mL fibrinogen and 5.5 IU/mL thrombin in PBS allow menstrual blood-derived mesenchymal stromal cell (MenSCs) adhesion and viability. The proposed coating method is easily reproducible and can be adapted to different cell types.

## 2. Results

### 2.1. Fibrin Sealant Components Characterization: Fibrin Clotting Test

To identify the most adequate conditions to coat the PP meshes, different concentrations of fibrinogen and thrombin in different buffers were tested and compared in terms of clotting capability. The step-by-step process to select buffers and concentrations of fibrinogen and thrombin is represented in Figure S1. 

For buffer selection, 1 and 5 mg/mL fibrinogen and 2.5 UI/mL thrombin solutions were prepared in Dulbecco’s Phosphate Buffered Saline (PBS), 2-(N-morpholino) ethanesulfonic acid (MES) buffered saline, citrate buffered saline, or Tris-buffered saline. With 1 mg/mL fibrinogen concentration, the consistency of the polymerized fibrin gel was too weak, regardless of the buffer used. With 5 mg/mL fibrinogen concentration, the consistency was harder with MES and PBS, while it was weak with citrate buffer and moderately sticky with Tris HCl buffer. Additionally, fibrinogen tended to precipitate in citrate buffer at pH 5.0. Hence, PBS and MES buffer were selected for further analyses. 

For concentrations selection, 1, 5, and 10 mg/mL fibrinogen solutions and 0.3, 1, 2.5, 5.5, 27.5, and 55 UI/mL thrombin solutions were prepared in PBS and combined to obtain different fibrin gel-like-coating. As it can be observed in Figure S2, with the highest concentrations of fibrinogen (5 and 10 mg/mL) and thrombin (27.5, and 55 IU/mL) the polymerization occurred immediately, and the resulted gel was hard. Hard gels were also obtained when 10 mg/mL fibrinogen were mixed with 5.5 and 2.5 IU/mL thrombin, taking 5 min to polymerize. With lower thrombin concentrations (1 and 0.3 IU/mL), 10 mg/mL fibrinogen generated tough gels and the time for polymerization was inversely proportional to thrombin concentration: 10 min for 1 IU/mL and 20 min for 0.3 IU/mL. 5 mg/mL fibrinogen produced a soft gel when mixed with 5.5, 2.5, and 1 IU/mL, being the polymerization quicker (10 min) for 5.5 and 2.5 IU/mL thrombin and slower (20 min) for 1 IU/mL. A weak gel was obtained with 5 mg/mL fibrinogen and 0.3 IU/mL thrombin in about 20 min. Similarly, with the lowest concentration of fibrinogen (1 mg/mL) and any of the proposed thrombin concentrations, very weak fibrin gels were obtained in more than 20 min (Figure S2). Taking the results of this test into consideration, 1 and 5 mg/mL and 2.5 and 5.5 IU/mL concentrations for fibrinogen and thrombin, respectively, were selected for further analyses.

### 2.2. Characterization of Plasma-Treated and Fibrin-Coated Meshes: Scanning Electron Microscopy Analysis

**Figure 1:**
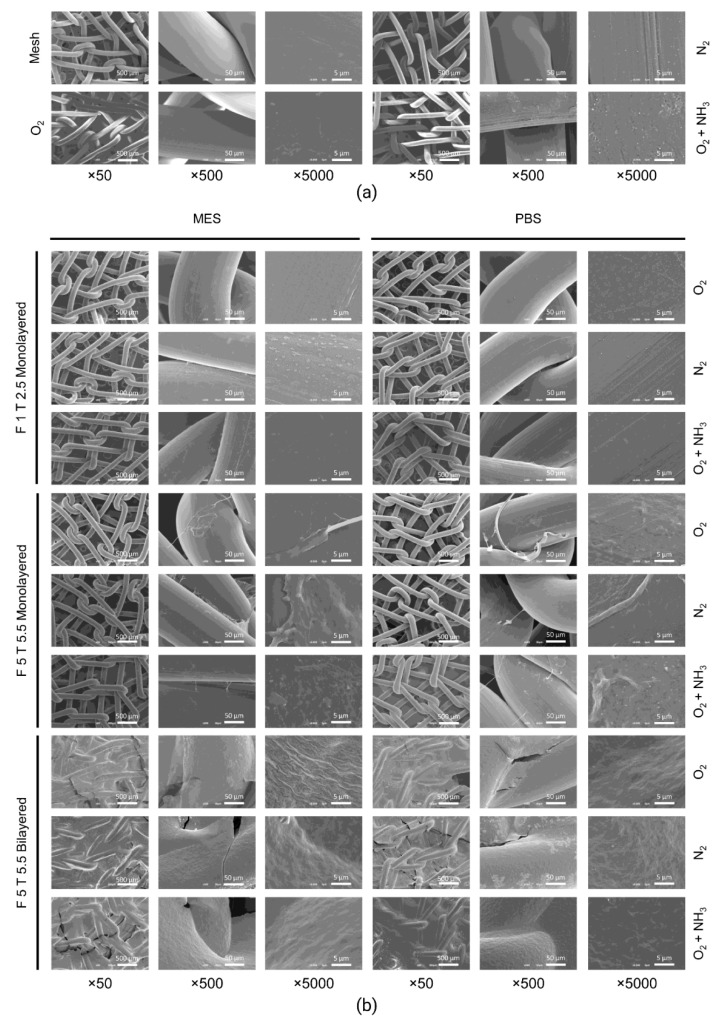
SEM micrographs of fibrin-coated surgical meshes with plasma treatment. (**a**) PP surgical meshes were treated with plasma (oxygen or nitrogen) and some of the oxygen plasma-treated meshes were immersed in ammonia. (**b**) Monolayered or bilayered fibrin coatings were performed with fibrinogen (F) and thrombin (T) from Tisseel^®^ (Baxter) in MES (2-(N-morpholino)ethanesulfonic acid buffered saline) or in PBS (phosphate-buffered saline) on plasma-treated meshes (oxygen, nitrogen or oxygen plasma-treated meshes immersed in ammonia). ×50, ×500, and ×5000 magnifications are shown. Fibrinogen and thrombin concentrations are expressed in mg/mL and IU (international units)/mL, respectively. F 1: fibrinogen 1 mg/mL; F 5: fibrinogen 1 mg/mL; N_2_, nitrogen plasma; O_2_, oxygen plasma; O_2_ + NH_3_, oxygen plasma + ammonia treatment; T 2.5: thrombin 2.5 IU/mL; T 5.5: thrombin 5.5 IU/mL.

The surface morphology of PP meshes (untreated or plasma-treated) and with or without fibrin coating, was investigated through scanning electron microscopy (SEM). The untreated PP meshes were characterized by a smooth surface, while oxygen and nitrogen plasma treatments changed slightly mesh surface, making it rougher. The biggest changes were observed with oxygen plasma followed by ammonia immersion, where crystals were deposited on the mesh surface (Figure 1a). 

Plasma-treated meshes were also coated with monolayered and bilayered fibrin coatings (Figure 1b). Monolayer fibrin coatings performed with the lowest fibrinogen and thrombin concentrations (1 mg/mL and 2.5 IU/mL, respectively) were almost unnoticeable, while higher concentrations resulted in thicker coatings. On the other hand, bilayered coatings were uniform and thicker.

**Figure 2:**
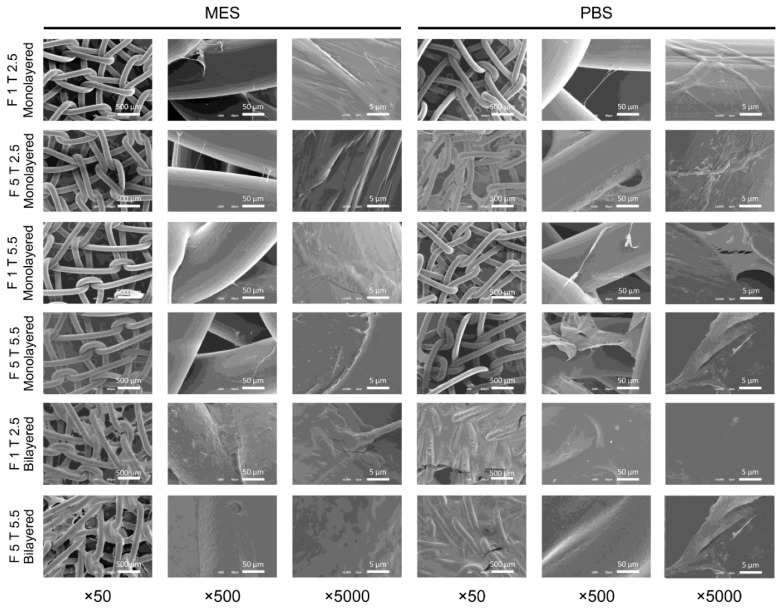
SEM micrographs of fibrin-coated surgical meshes without plasma treatment. Different combinations of fibrinogen (F) and thrombin (T) from Tisseel^®^ (Baxter) were diluted in MES or PBS and used to coat PP surgical meshes. ×50, ×500, and ×5000 magnifications are shown. Fibrinogen and thrombin concentrations are expressed in mg/mL and IU (international units)/mL, respectively. F 1: fibrinogen 1 mg/mL; F 5: fibrinogen 1 mg/mL; T 2.5: thrombin 2.5 IU/mL; T 5.5: thrombin 5.5 IU/mL.

Regarding the fibrin coating of untreated meshes, in general, when the two components of fibrin were diluted in PBS, the fibrin layers appeared slightly more homogeneous. Regardless of the concentration of thrombin (2.5 or 5.5 IU/mL), the lowest concentration of fibrinogen (1 mg/mL) seemed less effective than the highest (5 mg/mL) to provide a uniform coating. Increasing fibrinogen concentration to 5 mg/mL, the coating appeared thicker with the 2.5 IU/mL thrombin concentration than with 5.5 IU/mL thrombin and PBS as dilution buffer resulted in more homogeneous coatings than using MES. Bilayered fibrin coatings resulted in more uniform, regardless of fibrinogen and thrombin concentrations. This analysis suggested that monolayered fibrin coatings with the highest concentrations of thrombin and fibrinogen, as well as bilayered coatings, were necessary to uniformly cover PP surface (Figure 2). 

According to SEM results, none of the plasma and chemical treatments seemed to provide any additional benefit or detriment in terms of fibrin adhesion to the PP surface.

### 2.3. Mechanical Strength of Fibrin-Coated PP Meshes

**Figure 3:**
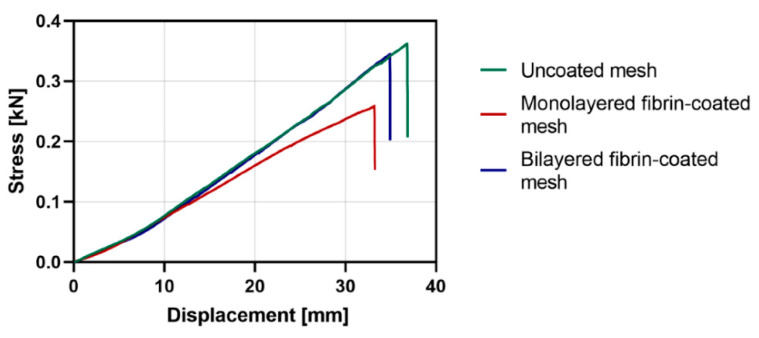
Stress-strain plots of the uniaxial tensile test. Stress vs. displacement curves for uncoated polypropylene (PP) surgical mesh, PP mesh with monolayered fibrin coating, and PP mesh with bilayered fibrin coating are shown. Monolayered and bilayered fibrin coatings were performed with 5 mg/mL fibrinogen and 5.5 IU/mL thrombin solutions in PBS.

The uniaxial tensile test revealed that uncoated PP meshes sustained a maximum load of 345.81 N before breaking, while the mesh with a monolayered fibrin coating (5 mg/mL fibrinogen and 5.5 IU/mL thrombin) sustained 258.37 N and the mesh with a bilayered fibrin coating (5 mg/mL fibrinogen and 5.5 IU/mL thrombin) sustained 344.8 N (Figure 3). Regarding elongation at break, the following results for uncoated, monolayered-coated, and bilayered-coated meshes were obtained: 123.36%, 110.53%, and 116.31%, respectively. Additionally, measured *E* was 35.07 MPa for uncoated meshes, 26.48 MPa for monolayered-coated meshes, and 35.22 MPa for bilayered-coated meshes. Uniaxial tensile test results are summarized in Table 1 and shown in Figure 3.

Overall, the slight differences in tensile strength, elongation at break, and *E* between uncoated and bilayered fibrin-coated meshes (5 mg/mL fibrinogen and 5.5 IU/mL thrombin) suggested that the bilayered coating was the most adequate for PP meshes in terms of preservation of mechanical parameters.

### 2.4. Viability and Adhesion of MenSCs Seeded on Fibrin-Coated PP Meshes

**Figure 4:**
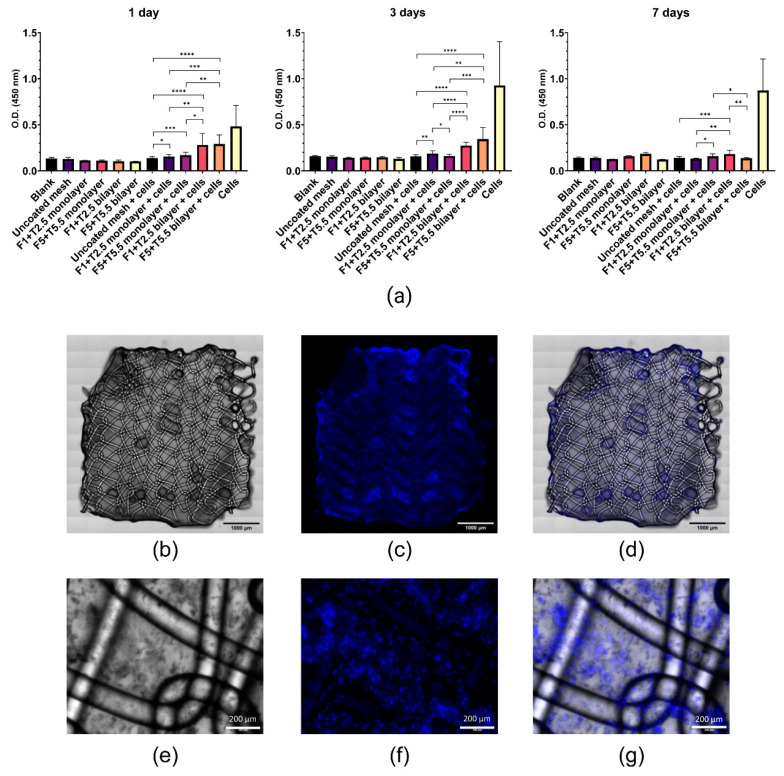
Viability and adhesion of menstrual blood-derived stromal cells (MenSCs) on fibrin-coated meshes. (**a**) A CCK-8 assay was performed 1, 3, and 7 days after cell seeding, measuring the absorbance at 450 nm. MenSCs seeded on uncoated meshes and MenSCs on a poly-D-Lysin-coated plate were used as negative and positive control, respectively (unpaired *t*-test, * *p* < 0.05, ** *p* < 0.01, *** *p* < 0.001, **** *p* < 0.0001). F 1: fibrinogen 1 mg/mL; F 5: fibrinogen 1 mg/mL; T 2.5: thrombin 2.5 IU/mL; T 5.5: thrombin 5.5 IU/mL; O.D.: optical density. MenSCs, stained with Hoechst 33342, were seeded on fibrin-coated PP meshes (bilayered fibrin coating with 5 mg/mL fibrinogen and 5.5 IU/mL thrombin), previously stained with Sudan Black B. Samples were visualized under a fluorescence microscope 1 day after cell seeding. The fibrin-coated mesh was visualized under visible light (**b**) and DAPI channel (**c**), then merged (**d**). Additionally, multiple images of selected fields were acquired at different focal planes along the z-axis (10x) and the two channels, visible (**e**) and DAPI (**f**) were merged (**g**). Scale bars represent 1 mm (**b**–**d**) or 200 μm (**e**–**g**).

Cell viability on fibrin-coated meshes was evaluated through a CCK-8 assay performed 1, 3, and 7 days after cell seeding. As shown in Figure 4a, absorbance read at 450 nm at day 1 was significantly higher in MenSCs seeded on all fibrin coatings compared with MenSCs on uncoated meshes, being more significant for the bilayered coatings (*p* < 0.0001) than for the monolayered coatings performed with the lowest (*p* = 0.264) and the highest (*p* = 0.001) fibrinogen and thrombin concentrations. A similar trend was observed at day 3 where MenSCs on bilayered coatings demonstrated significantly higher viability than MenSCs on uncoated meshes (*p* < 0.0001). However, while the viability of MenSCs was significantly higher on monolayered coatings with the lowest fibrinogen and thrombin concentrations (*p* = 0.003), no significant results were obtained with the monolayered coating with the highest concentrations, when compared with MenSCs on uncoated meshes. On the other hand, at day 7, only bilayered coatings with the lowest concentrations allowed MenSC viability to be significantly higher than MenSCs on uncoated meshes (*p* = 0.0007). In general, MenSC viability on bilayered-coated meshes was significantly higher than on monolayered coatings at all time points.

In addition, MenSCs seeded on fibrin-coated PP meshes (bilayered fibrin coating with 5 mg/mL fibrinogen and 5.5 IU/mL thrombin) were visualized under a fluorescence microscope 24 h after cell seeding. The overall visualization of the coated mesh sample revealed a uniform cell layer on the entire mesh surface (Figure 4b–d). Z-stacks of images at different focal planes showed that the mesh was encapsulated in the bilayered fibrin coating, being the cells almost all on the same focal plane (Figure 4e–g).

### 2.5. Phenotypic and Gene Expression Response of MenSCs to the Fibrin Coating

**Figure 5:**
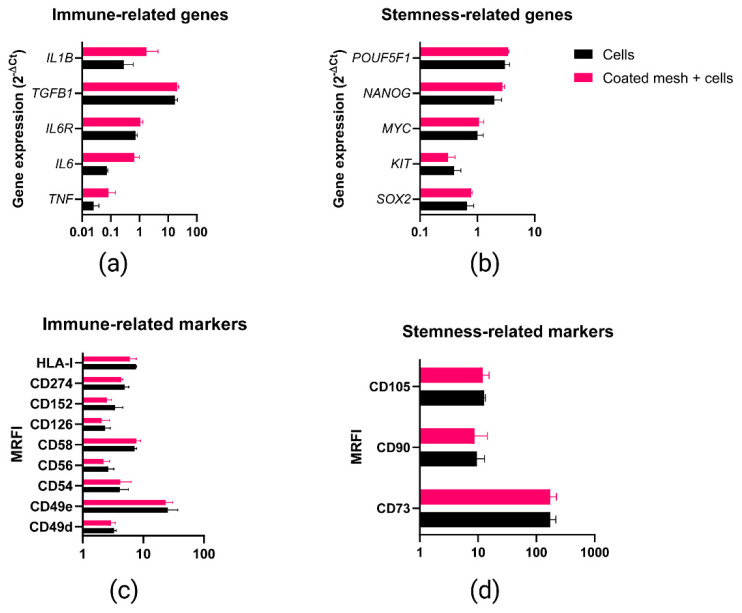
Gene expression and phenotypic marker analyses for Coated mesh + cell vs. Cells group. “Cells” refers to menstrual blood-derived stromal cells (MenSCs) seeded on uncoated PP meshes; “Coated mesh + cells” refers to MenSCs seeded on fibrin-coated PP meshes (bilayered fibrin coating performed with 5 mg/mL fibrinogen + 5.5 IU/mL thrombin solutions in PBS). Immune-related (**a**) and stemness-related (**b**) gene expression was evaluated through quantitative PCR (qPCR) analysis. Likewise, immune-related (**c**) and stemness-related (**d**) surface markers were analyzed through flow cytometry. Mann-Whitney test revealed no statistically significant differences between the two groups. Values along the x-axis are represented in logarithmic scale. MRFI: mean relative fluorescent intensity.

To further explore if the bilayered fibrin coating (5 mg/mL fibrinogen and 5.5 IU/mL thrombin) could trigger any change in MenSCs phenotypic and genetic profile, stemness (*POUF5F1*, *NANOG*, *MYC*, *KIT*, *SOX2*) and immune-related genes (*IL1B*, *TGFB1*, *IL6R*, *IL6*, *TNF*) were evaluated through qPCR. No statistically significant differences were found between MenSCs seeded on uncoated mesh vs. MenSCs seeded on bilayered fibrin-coated mesh (5 mg/mL fibrinogen and 5.5 IU/mL thrombin) (Figure 5a,b). Likewise, immune-related (CD49d, CD49e, CD54, CD56, CD58, CD126, CD152, CD274, HLA-I) and stemness (CD73, CD90, CD105) surface markers were evaluated through flow cytometry. The analysis of surface markers confirmed that the fibrin coating did not affect MenSC profile when seeded on a bilayered fibrin-coated mesh, as no statistically significant differences were found (Figure 5c,d). These results demonstrated that MenSC stemness was not negatively affected by the presence of the bilayered fibrin-coating. Similarly, according to the analyzed markers, no inflammatory response was triggered in MenSCs by the fibrin-coated meshes.

### 2.6. Proliferative Ability of In Vitro-Stimulated T Cells Co-Cultured with Fibrin-Coated Meshes Seeded with MenSCs

**Figure 6:**
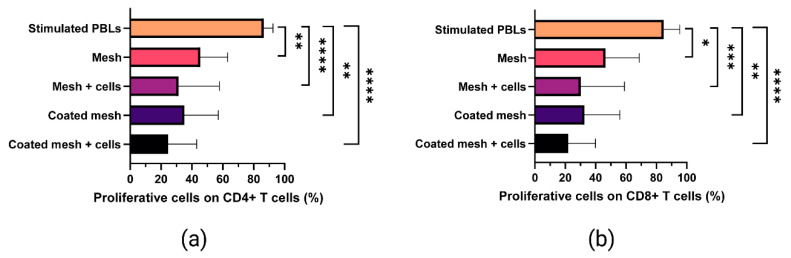
Proliferative ability of in vitro-stimulated T cells co-cultured with fibrin-coated meshes seeded with cells. Peripheral blood lymphocytes (PBLs) from three human donors were labeled with carboxyfluorescein succinimidyl ester (CFSE) and stimulated in vitro with T cell activation/expansion beads. During in vitro stimulation, PBLs were co-cultured with uncoated surgical meshes (Mesh), uncoated surgical meshes seeded with menstrual blood derived-stromal cells (MenSCs, n = 3) (Mesh + cells), fibrin-coated PP meshes (Coated mesh), and fibrin-coated PP meshes seeded with MenSCs (Coated mesh + cells). Coated meshes received a bilayered fibrin coating with 5 mg/mL fibrinogen + 5.5 IU/mL thrombin solutions in PBS. CFSE low cells (proliferative T cells) are presented in terms of percentage on CD4+ (**a**) and CD8+ (**b**) cells. Mean ± SD are represented. Mann-Withney tests allowed to compare each group with the positive control (stimulated PBLs without meshes and cells). * *p* < 0.05; ** *p* < 0.01; *** *p* < 0.001; **** *p* < 0.0001.

Carboxyfluorescein succinimidyl ester (CFSE)-labeled and in vitro stimulated PBLs were analyzed by flow cytometry in order to explore the potential effects of MenSCs seeded on fibrin-coated meshes on CD4+ and CD8+ T cell proliferation (Figure 6). CFSE low cells (proliferative T cells) were presented in terms of percentage on CD4+ (Figure 6a) and CD8+ (Figure 6b) cells. Statistically significant differences in terms of reduction of proliferation were found when CD4+ T cells were co-cultured with MenSCs seeded on uncoated meshes (*p* < 0.0001) and on bilayered fibrin-coated meshes (5 mg/mL fibrinogen and 5.5 IU/mL thrombin) (*p* < 0.0001), when compared to the positive control (in vitro stimulated PBLs). Similarly, MenSCs seeded on uncoated meshes (*p* = 0.0003) and on bilayered fibrin-coated meshes (*p* < 0.0001) reduced CD8+ T cell proliferation compared to the positive control. The fibrin coating itself (devoid of MenSCs) induced a significant reduction in CD4+ and in CD8+ T cell proliferation (*p* = 0.0091). Surprisingly, even uncoated meshes reduced significantly CD4+ (*p* = 0.0091) and CD8+ (*p* = 0.0364) T cell proliferation. 

## 3. Discussion

The paracrine potency is a remarkable property of MSCs and it has been widely demonstrated to enhance tissue repair [22] and to be involved in the immunomodulation of immune responses [23]. The synergic use of surgical mesh implantation and MSC-based therapies has been repeatedly proposed in the last decade, leading to improvements in vascularization, collagen deposition, tissue formation, macrophage response, and wound healing post hernia surgery [7]. Many types of MSCs have been seeded on surgical meshes, especially from adipose tissue and bone marrow [7]. We chose MSCs from menstrual blood for their notable potential for clinical applications, having a high proliferative rate and differentiation capacity [24]. Additionally, MenSC isolation is easy, painless, quick, cheap, and free of ethical concerns, compared to other MSC sources [24,25]. Even though MenSCs were proposed for the treatment of pelvic organ prolapse (POP) for their differentiation capacity towards muscle cells [26], from the best of our knowledge, they were never coupled to surgical meshes and used as a cell source for cellular therapy for hernia or POP treatment. Nevertheless, some authors have explored the effects of different meshes seeded or printed with MSCs from endometrial biopsies in in vitro and in vivo studies. These endometrial MSCs, seeded on polyamide [27], polyamide/gelatin [28,29,30], PLACL/gelatin nanofiber [31] meshes, or even 3D bio-printed with PCL scaffolds [32], demonstrated an overall immunomodulatory and regenerative effect in murine models of wound healing [28,29,30,32] and ovine models of POP [27]. According to the mentioned studies, cell therapy was vital to reducing some of the adverse effects following herniorrhaphy. However, the inertness and hydrophobicity of PP fibers make cell adhesion hard [33] and functionalizing PP meshes is highly necessary. Different types of chemical functionalization of non-absorbable meshes, like the ones based on sodium hydroxide [33], atomic layer deposition and vapor phase chemical grafting [34], and gas plasma [35], have been proposed to add functional groups on the non-polar PP surface. Typical plasma treatments with nitrogen are known to add carbonyl and amino groups to PP [36], while oxygen plasma is known to add hydroxyl, carboxylic acid, and aldehyde negative groups [37], leading to an increase in hydrophilicity, wettability, and biocompatibility of the treated synthetic material [38,39]. Hence, many authors have recently performed plasma surface functionalization of PP meshes to allow the adhesion of antimicrobial agents [40], antibiotics [41], other functional molecules, like betaine hydrochloride [42], or hydrogels [43]. The use of plasma treatment to allow the adhesion of the fibrin gel-like coating to PP mesh surface is particularly advantageous. Indeed, gels work as physical barriers between the viscera and peritoneum and help preventing postoperative adhesion [44]. Hence, in this study, we proposed a method to allow the adhesion of a fibrin gel-like coating to PP meshes that would also work as a vehicle for cell therapy. 

First, we proposed a multilayered fibrin coating for surgical PP meshes. Its optimization required the evaluation of different buffers and different concentrations of fibrinogen and thrombin from a commercial fibrin sealant. Four saline buffers with different pH were chosen taking into consideration the isoelectric points (pI) of human thrombin (pI = 7.0–7.6) [45] and human fibrinogen (pI = 5.8) [46], and also considering previous studies [47,48]. On the other hand, fibrinogen and thrombin concentrations to be tested were chosen according to previous studies [47,48,49,50,51,52] and to the datasheet of the commercial fibrin sealant Tisseel^®^ (Baxter) [53], that was used in this study. Our fibrin clotting test revealed that 1 and 5 mg/mL and 2.5 and 5.5 IU/mL concentrations for fibrinogen and thrombin, respectively, in MES buffer or PBS, guaranteed the formation of the most appropriated fibrin-like gels in terms of density and clotting time. To test the feasibility of our proposed method for a multilayered fibrin coating and to explore if plasma treatment could improve fibrin gel-like adhesion on the PP mesh, SEM was performed. Despite the plasma treatment, the fibrin coating, regardless of concentrations and buffers, seemed to adhere similarly to the untreated PP and to the plasma-treated reactive meshes. This result was confirmed when meshes were immersed in ammonia solution after oxygen plasma, which should have further increased PP reactivity, but did not seem to promote higher intermolecular interactions between the low-surface energy template (PP) and the fibrin coating. Hence, according to our findings, untreated meshes were used as templates for the following assays. 

Regarding the choice of the best dilution buffer for fibrin sealant components, SEM analysis showed that fibrinogen and thrombin dilutions in MES buffer seemed neither to improve, nor to worsen the performance of the fibrin coating in terms of adhesiveness or stability. On the other hand, according to our interpretation, the coatings in PBS resulted slightly more homogeneous. Dilutions of fibrinogen and thrombin from commercially available fibrin sealants have been proposed by different authors, especially to reduce setting time, bursting pressure, leaks, and dehiscence when fibrin sealants are used as surgical glues. Both components have shown to be functional when diluted in injectable water [54] and TRIS buffer [48], even though most of the authors preferred to dilute thrombin in 0.9 % sodium chloride solution [55] or balanced saline solution [56], leaving fibrinogen undiluted. Contrarily, Guillaume et al. [18] diluted the fibrinogen component in PBS leaving the thrombin undiluted. Calcium chloride has been stated to be necessary for the assembly of fibrin fibers [57], however, we did not add calcium chloride to any of the proposed buffers, being it already present in the thrombin preparation from Tisseel^®^ at a concentration of 36–44 μmol/mL [53]. So, considering the similar behavior of fibrin sealant components in PBS or MES buffer and that MES buffer is more used for plant cells [58], while PBS is a commonly used buffer for MenSC isolation, characterization, and differentiation [59] we continued our study diluting Tisseel^®^ components in PBS. 

Cell-based assays were performed to evaluate the behavior of MenSCs seeded on monolayered or bilayered fibrin coatings. Our cell viability assays demonstrated that MenSCs seeded on fibrin-coated meshes were significantly more metabolically active than MenSCs seeded on uncoated meshes, being particularly evident for bilayered coatings. This result suggests that a thicker fibrin coating is necessary for cell retention. A possible explanation of the fact that bilayered coatings were more effective in terms of cell retention is that fibrin coatings are prone to be detached and washed away by buffer or water washings, as we observed by SEM analysis. Here we hypothesize that thinner coatings are washed away more easily than thicker bilayered coatings. Repeated washings and medium changes may have made the coating (hence the seeded cells) detach for the weakening of hydrophobic interactions and this may explain the low cell viability measured 7 days after seeding. Another possible explanation of the decrease in cell viability observed 7 days after seeding is that a thicker film may have reduced the diffusion of nutrients and oxygen, necessary for cell survival and proliferation. Even though further in vitro and in vivo studies are necessary to elucidate if this issue may compromise the long-term in vivo effect of the implanted cells, we recommend seeding an adequate number of MSCs to cover the entire surface of the mesh and to incubate the cell-seeded surgical material for one day, then proceed to its implantation. Regardless, we may hypothesize that the MSCs on the functionalized mesh do not need to survive for a long time because, as soon as they encounter an inflamed microenvironment, like the one that results from a mesh surgery, they start to secrete soluble factors and extracellular vesicles that have the potential to modulate the exacerbated inflammatory reaction and to induce tissue regeneration [60,61]. As previously stated, this is just a speculation and only in vivo studies can confirm if a short viability of the locally administered stem cells can compromise their real efficacy. 

To further establish which concentrations of fibrinogen and thrombin should be used to get the most adequate bilayered fibrin coating, uniaxial tensile tests were performed. We observed that the bilayered coating with 5 mg/mL fibrinogen and 5.5 IU/mL thrombin preserved the mechanical strength of PP meshes more than the coating with lower concentrations of fibrin sealant components. Given that the advantage of using synthetic PP meshes lies in their robust mechanical properties, it is important to prevent a detrimental effect of the coating on mesh mechanical strength. To our knowledge, the effect of a fibrin coating on PP meshes has never been evaluated in terms of mechanical parameters, however, Wolf et al. highlighted that a coating with extracellular matrix did not affect the mechanical properties of explanted PP meshes after an in vivo study [17].

The adhesion of MenSCs on bilayered fibrin-coated meshes (with 5 mg/mL fibrinogen and 5.5 IU/mL thrombin) was then assessed through fluorescence labeling. It should be noted that many materials emit fluorescence signals that make the observation of the labeled cells challenging [62]. Hence, staining the fibrin-coated meshes with Sudan Black B before MenSC seeding was necessary to reduce the aforementioned autofluorescence, according to a previously suggested approach for PCL scaffolds [62]. According to our experiment, MenSCs were adherent to the fibrin-coated mesh, confirming the results from a previous study where keratinocytes, fibroblasts, and endothelial cells were demonstrated to attach, spread, and proliferate in the same fibrin sealant that was used for our coating [63]. Moreover, cells from SVF seeded on a PP mesh using fibrin sealant as a vehicle, were also observed at larger time points (21 days) revealing a proliferative activity of SVF cells and their capability of infiltrating the fibrin-like gel [18]. 

To be sure of the potential of the fibrin-coated meshes as a vehicle for cell therapy, we also investigated if the fibrin coating could trigger an inflammatory response in the seeded MenSCs, as well as any modification to their pluripotentiality. Our results showed that the fibrin gel-like coating where the cells were seeded, was harmless for the MenSCs and, in addition, it enhanced the therapeutic effect of our MSCs, as described earlier by other authors [64]. In our case, the synergistic antiproliferative effect of the fibrin sealant and the MSCs was demonstrated on activated T lymphocytes, suggesting that this coating method may significantly enhance tissue regeneration by modulating the immune response in injured tissues. Indeed, the beneficial effects of the local application of MSCs in a fibrin network have already been accomplished in various preclinical and clinical studies. For example, the local application of fibrin sealant and adipose tissue-derived MSCs was demonstrated to induce fistula healing in patients with Crohn’s disease [65]; bone marrow-derived MSCs in fibrin glue conduits reduced macrophage reaction and enhanced axonal regeneration after peripheral nerve injury [66]; furthermore, the epicardial placement of bone marrow-derived MSCs in fibrin sealant enhanced MSC engraftment in a rat ischemic cardiomyopathy model [67]. 

Further studies should elucidate if MSCs provide any additional benefit to the newest coating methods to counteract the adverse effects of PP mesh implantation, like bacterial nanocellulose [68], thermal atomic layer deposition [69], chlorhexidine or rifampicin-carboxymethylcellulose biopolymer gel [70], or zipper-like coatings based on tannic acid, Fe^3+^, and poly ((2-methacryloyloxyethyl phosphorylcholine)-(acrylic acid))-g-dopamine [71], among others. 

To conclude, despite the volume of research in the field, for patients that require soft tissue reinforcement, “nothing is certain but mesh and taxes”. A plethora of options are clinically available, but surgical mesh repair is still challenging and inevitably leads to adverse effects and relapse. The coating method proposed in this study may lead to the manufacturing of an off-the-shelf product for soft tissue reinforcement involving cell therapy. However, the production of the perfect mesh requires a multidisciplinary and translational approach. This in vitro proof of concept surely requires further functional and in vivo studies to improve the biological performance of our fibrin-coated PP meshes for future clinical uses. 

## 4. Materials and Methods

### 4.1. Optimization of the Fibrin Coating

The two components of the fibrin sealant Tisseel^®^ (Baxter, Deerfield, IL, USA, product number 1504516), hereinafter called “fibrinogen” and “thrombin” were used to optimize a coating for PP surgical meshes. Further information about composition and mechanism of action can be found in the package leaflet of Tisseel^®^ [53]. The graphical representation of the methodology, showing how concentrations and buffers for the fibrin coating were chosen for all the experiments, can be found in Figure S1.

To select the best buffer for thrombin and fibrinogen clotting, 1 and 5 mg/mL fibrinogen and 2.5 IU/mL thrombin solutions were prepared in PBS at pH 7.4; in 20 mM MES buffered saline (150 mM NaCl) at pH 6.5; in 50 nM citrate buffered saline (150 mM NaCl) at pH 5.0; and in 50 mM Tris-buffered saline (150 mM NaCl) at pH 8.0. All reagents used to prepare the aforementioned buffers were purchased from Sigma Aldrich, Merck (Darmstadt, Germany).

Afterward, different solutions of thrombin (0.3, 1, 2.5, 5.5, 27.5, and 55 IU/mL) and fibrinogen (1, 5, and 10 mg/mL) were prepared in PBS to test the best concentrations to obtain an adequate fibrin gel for mesh coating. The clotting test was performed by mixing 50 µL of fibrinogen solutions with 50 µL of thrombin solutions on the top of a Petri dish and letting the droplets dry at room temperature. For a more objective evaluation of hardness and time of polymerization of fibrin gels, a scoring system was established. For fibrin gel hardness, the following score was assigned: 0, no polymerization; 1, weak gel, easily broken with a pipette tip, a liquid content is released once broken; 2, soft gel, easily broken with a pipette tip, remains solid once broken; 3, tough gel, resistant to pressure, harder to break with a pipette tip, remains solid once broken; 4, hard gel, resistant to pressure, very hard to break with a pipette tip. For polymerization time, the following score was assigned: 0, no polymerization; 1, about 20 min; 2, about 10 min; 3, about 5 min; 4, immediate polymerization, a gel is formed as soon as the two components get in touch inside of the pipette tip.

### 4.2. Plasma Treatment of the PP Meshes

PP surgical meshes Assumesh^®^ standard (code AM1515, Assut Europe, Rome, Italy), with 90 g/m^2^ weight, were used throughout the study. The treatment was performed in an Atmospheric DBD (dielectric barrier discharge) Plasma Treatment System (Sigma Technologies International, Tucson, AZ, USA) equipped with a roll-to-roll apparatus with fully automated process control. PP meshes were clamped between the electrodes and a mixture of Ar and O_2_ gases or a mixture of Ar and N_2_ gases were used. Regardless of the type of gas, the plasma treatment was performed with the following conditions: atmospheric pressure; 10 m/min speed; 1500 W power; 4.9 Amp intensity of electrodes; 7500 kV difference in potential; 3 mm electrode separation; 100 set point and 439 flow SCCM (gas 1 on); 45 set point and 448 flow SCCM (gas 2 off). Following the treatment, part of the meshes treated with O_2_ plasma was immediately submerged in ammonia 25% (*v/v*) solution. 

### 4.3. Fibrin Coating of PP Meshes

**Figure 7:**
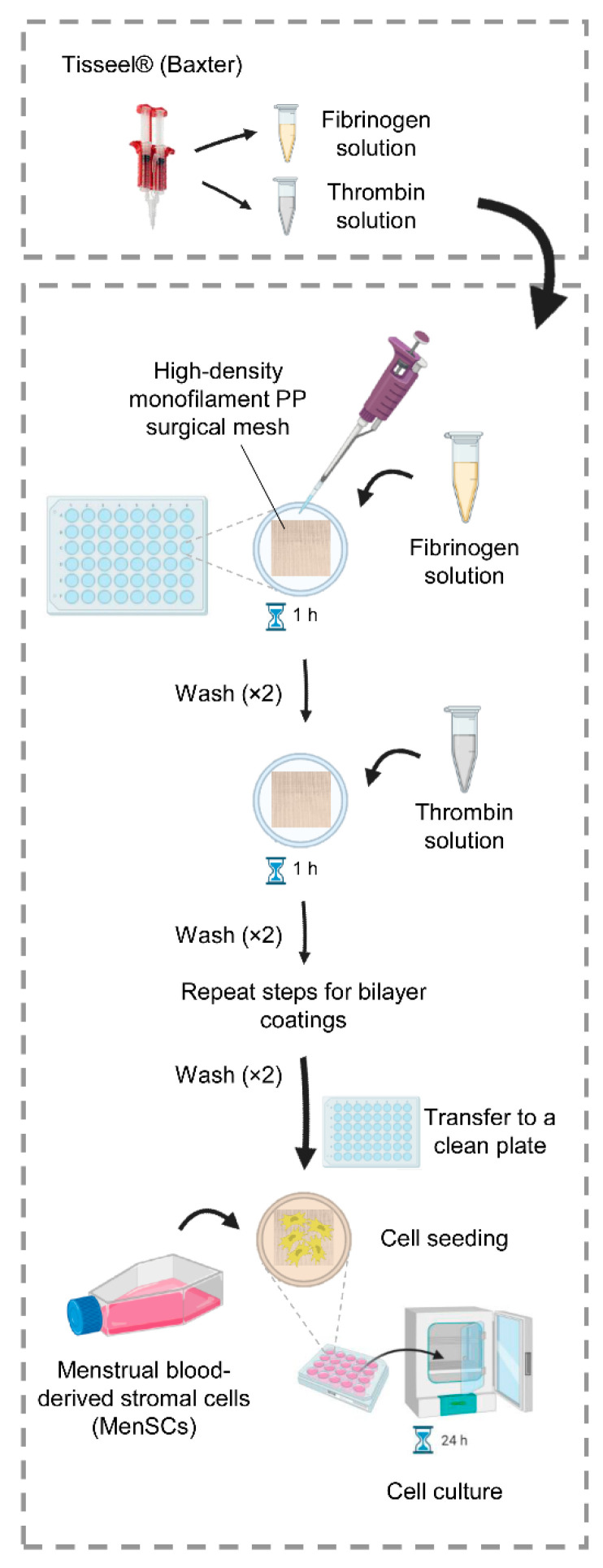
Fibrin coating process of polypropylene (PP) surgical meshes. For each experiment, fibrinogen and thrombin solutions were prepared in PBS or MES buffer. PP surgical meshes, deposited on the bottom of ultra-low attachment multiwell plates, were covered by fibrinogen solutions for 1 h, then rinsed with the proper buffer. Afterward, samples were covered with thrombin solutions for 1 h and rinsed with the proper buffer to obtain monolayered coated meshes. To get bilayered coated meshes, the coating procedure was repeated. For cell seeding, fibrin-coated meshes were transferred to a clean plate and menstrual blood-derived stromal cells were seeded ensuring a complete covering of the mesh and cultured in a cell incubator for 24 h, unless otherwise indicated.

PP meshes, with (plasma-treated meshes) and without (untreated meshes) plasma treatment, were cut with a paper cutter and deposited on the bottom of ultra-low attachment 96-well plates (Corning Costar, Merck, Darmstadt, Germany). 1 and 5 mg/mL fibrinogen solutions, and 2.5 and 5.5 UI/mL thrombin solutions were prepared in PBS or MES buffer. Monolayered and bilayered fibrin coatings on PP surgical meshes were performed combining different fibrinogen and thrombin concentrations as follows: the meshes were immersed in fibrinogen solutions for 1 h, then they were rinsed with the proper buffer (PBS or MES) and activated with thrombin solutions for 1 h. After thrombin activation, meshes were rinsed with the proper buffer (PBS or MES) to obtain monolayered fibrin-coated meshes. For bilayered fibrin-coated meshes, the samples were turned upside down and the coating procedure was repeated as in the previous steps. The coating process is shown in Figure 7. Fibrin-coated meshes were stored at 4 °C overnight, then processed for SEM analysis. Morphology and distribution of the fibrin coating network on PP meshes were studied with a JSM-6010 LV (JEOL, Japan) SEM instrument. All the samples were sputtered with a conductive gold layer, using a sputter coater SC502 (Fison instruments, Farnborough, UK). 

### 4.4. Uniaxial Tensile Test

The mechanical properties of PP meshes with monolayered and bilayered coatings with fibrinogen (5 mg/mL) and thrombin (5.5 IU/mL) solutions in PBS, were also studied using an Instron 5965 series Universal Testing System (Instron, Norwood, MA, USA) with a load cell of 5 kN. A schematic illustration of the samples, with their dimensions, is shown in Figure S3. PP meshes were secured in the grips of the instrument and uniaxial tensile tests were performed to failure at a strain rate of 100 mm/s. Maximum breaking load, elongation at break, and Young modulus (*E*) were expressed in Newtons (N), percentage (%), and Megapascals (MPa), respectively.

### 4.5. Cell Seeding and CCK-8 Assay

Previously isolated and cryopreserved MenSCs were used. All menstrual blood donors provided written informed consent to participate in the study, approved by the Jesús Usón Minimally Invasive Surgery Centre Research Ethics Committee. Briefly, MenSCs from three healthy women were isolated, expanded, and characterized as in previous studies [60,61,72]. For cellular studies, PP meshes without plasma treatment were cut under a laminar flow hood with sterile scalpel and scissors, then deposited on the bottom of ultra-low attachment 48-well non-treated multidishes (Nunc, Thermo Fisher Scientific, Waltham, MA, USA). Monolayered and bilayered fibrin coatings were performed with the lowest (1 mg/mL and 2.5 IU/mL) and the highest (5 mg/mL and 5.5 IU/mL) concentrations of fibrinogen and thrombin, respectively, on PP meshes without plasma treatment, following the above-mentioned steps. Subsequently, the fibrin-coated meshes were transferred into clean ultra-low attachment 48-well plates and seeded with 500 µL suspension of MenSCs, at passages 10–15, in Dulbecco’s Modified Eagle’s Medium (DMEM, Corning™, Thermo Fisher Scientific, Waltham, MA, USA) supplemented with 10% of fetal bovine serum (FBS, Gibco, Thermo Fisher Scientific, Waltham, MA, USA), 1% penicillin/streptomycin (Lonza, Basel, Switzerland), and 1% glutamine (Lonza, Basel, Switzerland), at a density of 1 × 10^5^ cells/mesh. As negative controls, uncoated meshes with or devoid of MenSCs, and fibrin-coated meshes devoid of cells were used. Furthermore, to provide positive controls of cell viability and adhesion, some wells of the ultra-low attachment 48-well plates were coated with poly-D-lysin (Merck, Darmstadt, Germany) 10 mg/L for 1 h, then washed with PBS and MenSCs were seeded at a density of 1 × 10^5^ cells/well. Each sample was prepared in triplicate. All samples were incubated at 37 °C with 5% of CO_2._ CCK-8 assay was performed after 1, 3, and 7 days of cell incubation. At these time points, cell culture media were removed from each well, the samples were washed once with PBS and transferred into new clean 48-well plates. The wells were filled with DMEM without phenol red (Gibco, Thermo Fisher Scientific, Waltham, MA, USA), supplemented with 10% of fetal bovine serum (FBS), 1% penicillin/streptomycin, and 10% CCK-8 solution (Boster Biological Technology, Pleasanton, CA, USA). Plates were left in the incubator for 1 h, then 100 µL of supernatants were transferred to 96-well plates. The absorbance at 450 nm was recorded in a Synergy™ Mx Microplate Reader (Biotek, Winooski, VT, USA).

### 4.6. Hoechst Staining and Fluorescence Microscopy

MenSC adhesion on the top of fibrin-coated PP meshes was verified by fluorescence microscopy. Bilayered coatings with the highest (5 mg/mL and 5.5 IU/mL) concentrations of fibrinogen and thrombin, respectively, were performed on PP meshes without plasma treatment. To reduce autofluorescence and light refraction, 1% Sudan Black B (SBB) solutions (Alfa Aesar, Thermo Fisher Scientific, Waltham, MA, USA) (10 mg/mL) were prepared in 70% (*v/v*) ethanol. The solution was then sterilized with a 0.22 μm filter and diluted to 0.3% (*v/v*) SBB solutions in 70% (*v/v*) ethanol. Fibrin-coated meshes were immersed in SBB solutions overnight at room temperature. Before cell seeding, SBB-stained fibrin-coated meshes were washed twice with PBS and left to dry in the flow chamber at room temperature. MenSCs (n = 1) at passage 16 were trypsinized, centrifuged, and stained in a 1 μL/mL Hoechst 33342 (Invitrogen, Thermo Fisher Scientific, Waltham, MA, USA) stock solution in PBS for 10 min protected from light. Cells were washed in PBS and 2 × 10^5^ cells/well were resuspended in 500 μL DMEM + 10% FBS and 1% penicillin/streptomycin, seeded on fibrin-coated meshes in 48-well Non-Treated Multidishes (Nunc, Thermo Fisher Scientific, Waltham, MA, USA), and cultured for 24 h. The samples were carefully mounted between two slides, securing the extremities with adhesive tape. Pictures were taken with an Evos FL Auto 2 Cell Imaging System (Thermo Fisher Scientific, Waltham, MA, USA). For an overall visualization of the mesh sample, a scan protocol was created. Additionally, multiple images of selected fields were acquired at different focal planes along the z-axis (10×). Images were stacked with Fiji-Image J [73] and z projects were created (“Sum slices” for the visible light channel, “Max intensity” for the blue channel). The background was subtracted to reduce light refraction and brightness and contrast were adjusted if necessary. Finally, the visible and DAPI channels were merged. 

### 4.7. Phenotypic and Gene Expression Analyses

To clarify if the fibrin coating affects the phenotypic and gene expression profile of MenSCs, bilayered coatings with the highest (5 mg/mL and 5.5 IU/mL) concentrations of fibrinogen and thrombin, respectively, were performed on PP meshes without plasma treatment. 1 × 10^5^ MenSCs (n = 3) at passages 10–12 were seeded on the top of the coated meshes and incubated 24 h. The same number of MenSCs from the three lines were seeded on 24-well cell culture treated plates (BioLite, Thermo Fisher Scientific, Waltham, MA, USA) as control.

For the phenotypic analysis, and cells were detached with a 0.25% trypsin solution from the meshes and the plate wells. The detached cells were resuspended in PBS containing 2% FBS and stained for 30 min at 4 °C with the appropriate concentrations of the following human fluorescent-labeled monoclonal antibodies for stemness and immune-related markers: CD49d, CD54, CD90, CD105, CD152 (AbD Serotec, Bio-Rad, Hercules, CA, USA), CD49e, CD56, CD73, CD126, HLA-I (BD Pharmingen Inc., San Diego, CA, USA), CD58 (Diaclone, Besançon, France), and CD274 (eBioscience, Thermo Fisher Scientific, Waltham, MA, USA). Isotype-matched antibodies were be used as negative controls. The stained cells were washed, resuspended in PBS, and flow cytometry was performed by using a FACSCalibur™ Flow Cytometry System (BD Biosciences, San Jose, CA, USA). The CellQuest software (BD Biosciences, San Jose, CA, USA) was used to analyze the cells and the mean relative fluorescence intensity was be calculated by dividing the mean fluorescent intensity (MFI) by the MFI of its negative control. 

For gene expression analyses, total RNA from the detached cells was isolated with a mirVana™ miRNA Isolation Kit (Life Technologies, Thermo Fisher Scientific, Waltham, MA, USA) according to the manufacturer’s instructions. An Implen NanoPhotometer™ spectrophotometer (Fisher Scientific, Waltham, MA, USA) was used to evaluate RNA quality and concentration. A 260/280 nm absorbance ratio between 1.94 and 2.02 was measured in all RNA samples. Reverse transcription reactions were performed with iScript Reverse Transcription Supermix (BioRad, Hercules, CA, USA) according to the manufacturer’s instructions. For each sample, 15 ng cDNA were amplified in quantitative Real-Time PCR (qPCR) reactions with TaqMan Fast Advanced Master Mix (Applied Biosystems, Thermo Fisher Scientific, Waltham, MA, USA) and TaqMan^®^ Gene Expression Assays (Applied Biosystems, Thermo Fisher Scientific, Waltham, MA, USA) for the following stemness and immune-related genes: *ARG1* (Hs00163660_m1), *IL1B* (Hs01555410_m1), *IL6* (Hs00174131_m1), *IL6R* (Hs00174131_m1), *IL10* (Hs00961622_m1), *KIT* (Hs00174029_m1), MYC (Hs00153408_m1), *NANOG* (Hs02387400_g1), *NOS2* (Hs01075529_m1), *POUF5F1* (Hs04260367_gh), *SOX2* (Hs01053049_s1), *TGFB1* (Hs00998133_m1), and *TNF* (Hs00174128_m1). cDNA amplification reactions were performed in duplicate by using QuantStudio 3 System (Applied Biosystems, Thermo Fisher Scientific, Waltham, MA, USA) with the following thermal cycling conditions: 50 °C for 2 min, 95 °C for 2 min, and then 40 cycles of 95 °C for 1 s and 60 °C for 20 s. qPCR products were then quantified by fluorescent method using the 2^−ΔCt^ expression [74], normalizing against *TBP* gene (Hs00427620_m1). Lack of DNA contamination was shown by including duplicate no template control samples for each gene in the experiment.

### 4.8. Immunomodulatory Assay

To determine the immunomodulatory potential of fibrin-coated PP meshes on in vitro stimulated peripheral blood lymphocytes (PBLs), bilayered coatings with the highest (5 mg/mL and 5.5 IU/mL) concentrations of fibrinogen and thrombin, respectively, were performed on PP meshes without plasma treatment. 1 × 10^5^ MenSCs from 3 different donors were seeded on the top of uncoated and coated meshes and incubated for 24 h. PBLs, previously isolated from healthy donors (n = 3) and cryopreserved as in Blázquez et al., 2019 [75], were thawed in 37 °C water bath, washed and cultured in Roswell Park Memorial Institute (RPMI) medium 1640 (Biowest, Nuaillé, France) supplemented with 1% penicillin/streptomycin (Lonza, Basel, Switzerland), and 5% FBS (Gibco, Thermo Fisher Scientific, Waltham, MA, USA) overnight. The cells were centrifuged and pellets were stained with 0.5 μL/mL CellTrace™ CFSE (Thermo Fisher Scientific, Waltham, MA, USA) in PBS for 15 min at 37 °C. To remove dye remnants, PBLs were centrifuged, resuspended in supplemented RPMI-1640 medium, and left in a cell incubator for 30 min. Afterward, stained PBLs were centrifuged again, counted, and their concentration was adjusted to 3 × 10^6^ cells/mL. The PBLs were stimulated with a T cell activation/expansion kit (Miltenyi Biotec Inc, San Diego, CA, USA), according to the manufacturer’s recommendations and 100 μL of the PBLs suspension was seeded per well in 48-well plates. A total of 400 μL of supplemented RPMI-1640 medium was added per well and fibrin-coated PP meshes with MenSCs were completely immersed in the medium containing the stained and stimulated PBLs. MenSC-seeded meshes were co-cultured with stimulated PBLs for 4 days in a cell incubator. PBLs in suspension were collected from wells and analyzed by flow cytometry as reported in the previous section. Fluorescence-labeled human monoclonal antibodies against CD4 and CD8 (BD Biosciences, San Jose, CA, USA) were used. Stimulated PBLs without meshes were used as positive controls. The percentage of CFSE-low fluorescence intensity cells on gated CD4+ and CD8+ T cells was used to calculate the percentage of proliferative lymphocytes. 

### 4.9. Statistical Analysis

Shapiro-Wilk and D’Agostino & Pearson tests were used to assess normal and lognormal distribution of variables. For CCK-8 assay, an unpaired *t*-test was applied. For qPCR and flow cytometry analyses, Mann-Whitney test was used. The data were considered statistically significant at *p* values < 0.05. All the statistical tests were performed using the software GraphPad Prism 9.2.0 (GraphPad Software, San Diego, CA, USA).

## Figures and Tables

**Table 1:** Uniaxial tensile test results of uncoated and fibrin-coated polypropylene surgical mesh. Monolayered and bilayered fibrin coatings were performed with 5 mg/mL fibrinogen and 5.5 IU/mL thrombin solutions in PBS. For each condition, breaking load, elongation at break, and Young modulus were calculated following mechanical testing.

	Breaking Load [N]	Elongation at Break [%]	Young Modulus (*E*) [MPa]
Uncoated meshes	345.81	123.36	35.07
Monolayered coatings	258.37	110.53	26.48
Bilayered coatings	344.8	116.31	35.22

## Supplementary Materials

The following are available online at https://www.mdpi.com/article/10.3390/ijms222413385/s1.
