# Supplementary material for: A Fibrin Coating Method of Polypropylene Meshes Enables the Adhesion of Menstrual Blood-Derived Mesenchymal Stromal Cells: A New Delivery Strategy for Stem Cell-Based Therapies
Source: Int J Mol Sci. 2021 Dec 13;22(24):13385. doi: 10.3390/ijms222413385 (PMC8706515; doi:10.3390/ijms222413385)
Supplement: Supplementary file 1 [file ijms-22-13385-s001.zip › ijms-1494602-supplementary.pdf]

International Journal of *Molecular Sciences*

MDPI

Supplementary Materials

A Fibrin Coating Method of Polypropylene Meshes Enables the Adhesion of Menstrual Blood-derived Mesenchymal Stromal Cells: a New Delivery Strategy for Stem Cell-based Therapies

Federica Marinaro, Joana M. Silva, Alexandre A. Barros, Ivo M. Aroso, Juan C. Gómez-Blanco, Isaac Jardin, Jose J. Lopez, María Pulido, María Ángeles de Pedro, Rui L. Reis, Francisco Miguel Sánchez-Margallo, Javier G Casado and Esther López

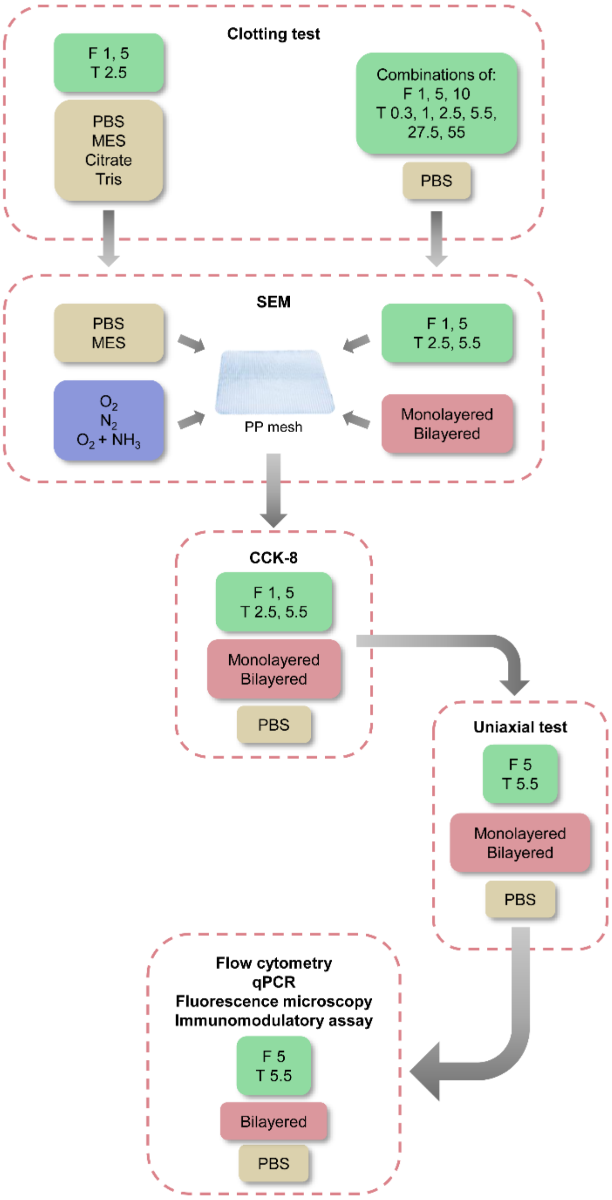

Figure S1: Graphical representation of the methodology. To choose the best fibrin coating for polypropylene surgical meshes, first, different combinations of fibrinogen (F) and thrombin (T) from Tisseel® (Baxter) (green

boxes) were diluted in different saline buffers (beige boxes) and selected in terms of clotting time and hardness. Second, surgical meshes underwent different plasma and chemical treatments (violet box). Monolayered or bilayered (burgundy boxes) coatings with the selected concentrations from the previous clotting tests were performed and analyzed through scattered electron microscopy (SEM). Third, biological and mechanical assays were carried out to furtherly select the best concentration and number of layers to coat PP meshes. Finally, the behavior of cells seeded on fibrin-coated meshes was evaluated through phenotypic, molecular, microscopy-based and immunomodulatory assays. Fibrinogen (F) and thrombin (T) concentrations are expressed in mg/ml and IU (international units)/ml, respectively. Scattered lines represent the assays that allowed the selection of the best concentrations, dilution buffer and type of coating. Citrate: citrate buffered saline; F: fibrinogen from Tisseel® (Baxter); MES: 2-(N-morpholino)ethanesulfonic acid buffered saline; PBS: phosphate buffered saline; qPCR: quantitative PCR; SEM: scanning electron microscopy; T: thrombin from Tisseel® ; Tris: Tris buffered saline.

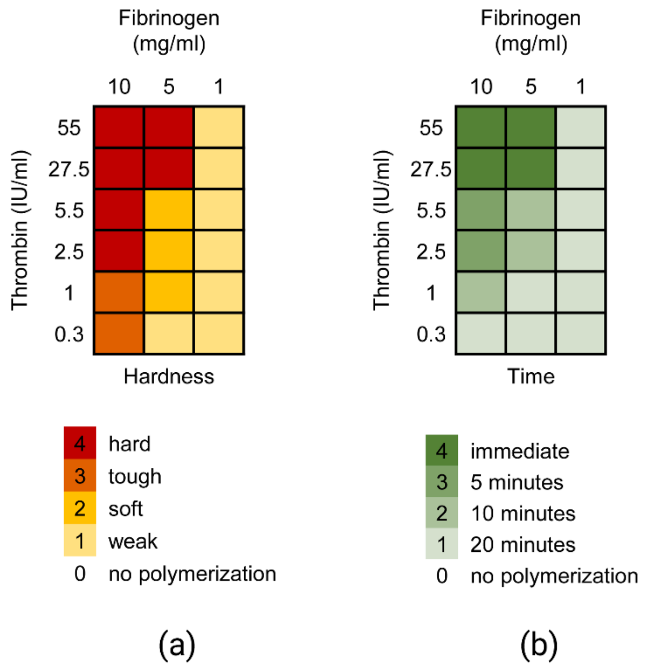

Figure S2: Fibrin clotting test. Fibrinogen and thrombin solutions at different concentrations were mixed and a scoring system was established for an objective evaluation of the fibrin gels. (a) Gel hardness was described with a scoring system from 0 to 4, corresponding to different gel textures from no polymerized to hard, respectively. (b) The polymerization time was described with a scoring system from 0 to 4, corresponding to increasing times from no polymerized to immediate, respectively.

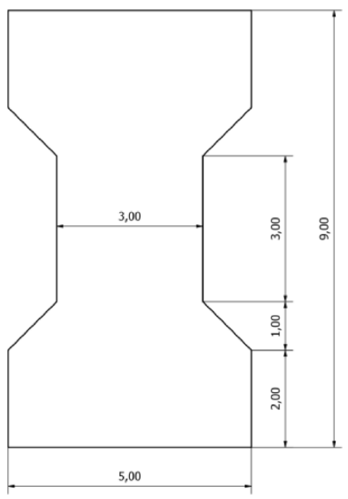

Figure S3: Dumbbell shape probe used for uniaxial tensile test. All dimensions are in cm.
